# The PDB database is a rich source of alpha-helical anti-microbial peptides to combat disease causing pathogens

**DOI:** 10.12688/f1000research.5802.2

**Published:** 2015-06-16

**Authors:** Sandeep Chakraborty, My Phu, Tâmara Prado de Morais, Rafael Nascimento, Luiz Ricardo Goulart, Basuthkar J. Rao, Bjarni Asgeirsson, Abhaya M. Dandekar

**Affiliations:** 1 Plant Sciences Department, University of California, Davis, CA, 95616, USA; 2 Department of Biological Sciences, Tata Institute of Fundamental Research, Homi Bhabha Road, Mumbai, 400 005, India; 3 Institute of Agricultural Sciences, Federal University of Uberlandia, Av. Amazonas, Bloco 2E, Campus Umuarama, Uberlandia, MG, Brazil; 4 Institute of Genetics and Biochemistry, Federal University of Uberlandia, Av. Amazonas, Bloco 2E, Campus Umuarama, Uberlandia, MG, Brazil; 5 Science Institute, Department of Biochemistry, University of Iceland, Dunhaga 3, IS-107 Reykjavik, Iceland

**Keywords:** PDB database, Ebola, alpha-helical antimicrobial peptides, SCALPEL

## Abstract

The therapeutic potential of *α*-helical anti-microbial peptides (AH-AMP) to combat pathogens is fast gaining prominence. Based on recently published open access software for characterizing *α*-helical peptides (PAGAL), we elucidate a search methodology (SCALPEL) that leverages the massive structural data pre-existing in the PDB database to obtain AH-AMPs belonging to the host proteome. We provide *in vitro* validation of SCALPEL on plant pathogens ( *Xylella fastidiosa*, *Xanthomonas arboricola* and *Liberibacter crescens*) by identifying AH-AMPs that mirror the function and properties of cecropin B, a well-studied AH-AMP. The identified peptides include a linear AH-AMP present within the existing structure of phosphoenolpyruvate carboxylase (PPC20), and an AH-AMP mimicing the properties of the two *α*-helices of cecropin B from chitinase (CHITI25). The minimum inhibitory concentration of these peptides are comparable to that of cecropin B, while anionic peptides used as control failed to show any inhibitory effect on these pathogens. Substitute therapies in place of conventional chemotherapies using membrane permeabilizing peptides like these might also prove effective to target cancer cells. The use of native structures from the same organism could possibly ensure that administration of such peptides will be better tolerated and not elicit an adverse immune response. We suggest a similar approach to target Ebola epitopes, enumerated using PAGAL recently, by selecting suitable peptides from the human proteome, especially in wake of recent reports of cationic amphiphiles inhibiting virus entry and infection.

## Introduction

The abundance of alpha helical (AH) structures present within proteins bears testimony to their relevance in determining functionality ^1^. AHs are key components in protein-protein interaction interfaces ^2^, DNA binding motifs ^3^, proteins that permeate biological membranes ^4^, and anti-microbial peptides (AMP) ^5, 6^. Not surprisingly, these AHs are the targets for antibody binding ^7, 8^ and therapeutic agents ^9^. These therapies in turn use AH peptides against both viral ^10– 12^ and bacterial pathogens ^13^.

Some AHs have unique characteristics, which are strongly correlated to their significance in the function of a protein ^7^. For example, hydrophobic residues aligned on one surface (characterized by a hydrophobic moment ^14^), is critical for virus entry into host cells ^15^, and in the permeabilizing abilities of AH-AMPs ^16^. Often, AHs have cationic residues on the opposite side of the hydrophobic surface, which helps them target bacterial membranes ^17, 18^. We have previously implemented known methods ^19^ of evaluating these properties, and provided this as open source software (PAGAL) ^20^. PAGAL was used to characterize the proteome of the Ebola virus ^7^, and to correlate the binding of the Ebola protein VP24 ^21^ to human karyopherin ^22^ with the immune suppression and pathogenicity mechanisms of Ebola and Marburg viruses ^23^.

Plant pathogens, like *Xylella fastidiosa* (Xf) ^24^, *Xanthomonas arboricola* (Xa) ^25^ and *Liberibacter crescens* (Lc) ^26^ are a source of serious concern for economic ^27^ and humanitarian reasons ^28^. Specifically, we have been involved in developing novel strategies to counter the Pierce’s disease causing Xf, having previously designed a chimeric protein with anti-microbial properties that provides grapevines with enhanced resistance against Xf ^29^. Cecropin B (CECB) is the lytic component of this chimeric protein ^30, 31^. However, the non-nativeness of CECB raises concerns regarding its viability in practical applications ^32^.

In an effort to replace CECB with an equivalent peptide from the grapevine/citrus genome, we present a design methodology to select AH-AMPs from any given genome - **S**earch **c**haracteristic **alp**ha h **el**ical peptides in the PDB database and locate it in the genome ( **SCALPEL**). CECB consist of two AHs, joined by a small loop. The N-terminal AH is cationic and hydrophobic, while the C-terminal AH consists of primarily hydrophobic residues. Characterizing all available AHs from plant proteins in the PDB database allowed us to identify a peptide with a large hydrophobic moment and a high proportion of positively charged residues, present in both grapevine and citrus (our organisms of interest), mirroring the linear cationic CECB N-terminal AH. One such match was a twenty residue long AH from phosphoenolpyruvate carboxylase in sunflower ^33^. The sequence of this peptide was used to find homologous peptides in the grapevine and citrus genome (PPC20). Subsequently, we used the SCALPEL algorithm to detect two contiguous AHs connected with a loop, mirroring the properties of CECB in a chitinase (CHITI25) from *Nicotiana tobaccum* (PDBid:3ALG) ^34^. Subsequently, we demonstrate through bioassay experiments that PPC20 from the grapevine and citrus genome, and CHITI25 from the tobacco genome, inhibit *Xf*, *Xa* and *Lc* growth. The minimum inhibitory concentration of these peptides are comparable to that of CECB, while anionic peptides used as controls failed to show any inhibitory effect with these pathogens. Further, we observed variation in the susceptibility of the pathogens to these peptides.

## Materials and methods

### *In silico* 

The PDB database was queried for the keyword ‘plants’, and proteins with the exact same sequences were removed. This resulted in a set of ~2000 proteins (see list.plants.txt in Dataset 1). These proteins were analyzed using DSSP ^35^ to identify the AHs, and AHs with the same sequence were removed. This resulted in ~6000 AHs (see ALPHAHELICES.zip in Dataset 1). PAGAL was applied to this set of AHs (see RawDataHelix.txt in Dataset 1). This data was refined to obtain peptides with different characteristics. We also computed the set of all pairs of AHs that are connected with a short (less than five residues) loop (see HTH in Dataset 1). This set is used to extract a pair of AHs, such that one of them is cationic with a large hydrophobic moment, while the other comprises mostly of hydrophobic residues. The PAGAL algorithm has been detailed previously ^20^. Briefly, the Edmundson wheel is computed by considering a wheel with centre (0,0), radius 5, first residue coordinate (0,5) and advancing each subsequent residue by 100 degrees on the circle, as 3.6 turns of the helix makes one full circle. We compute the hydrophobic moment by connecting the center to the coordinate of the residue and give it a magnitude obtained from the hydrophobic scale (in our case, this scale is obtained from Jones *et al.* ^19^). These vectors are then added to obtain the final hydrophobic moment. The color coding for the Edmundson wheel is as follows: all hydrophobic residues are colored red, while hydrophilic residues are colored in blue: dark blue for positively charged residues, medium blue for negatively charged residues and light blue for amides. All protein structures were rendered by PyMol ( http://www.pymol.org/). The sequence alignment was done using ClustalW ^36^. The alignment images were generated using Seaview ^37^. Protein structures have been superimposed using MUSTANG ^38^.

### *In vitro* 

Synthesized chemical peptides were obtained from GenScript USA, Inc. The protein molecular weight was calculated per peptide then diluted to 2000 *µ*M or 3000 *µ*M stock solutions with phosphate buffered saline. Stock solutions were stored in -20°C and thawed on ice before use.

Using the stock solutions, we made dilute solutions of 300 *µ*M, 250 *µ*M, 200 *µ*M, 150 *µ*M, 100 *µ*M, 75 *µ*M, 50 *µ*M, 30 *µ*M, 25 *µ*M, and 10 *µ*M to a final volume of 100 *µ*l of phosphate buffered saline. Dilute peptide solutions were stored in -20°C and thawed on ice before use.

*Xylella fastidosa* 3A2 (PD3) ^39^, *Xanthomonas arboricola* 417 (TYS) ^40^, and *Liberibacter crescens* BT-1 (BM7) ^41^ media were prepared and autoclaved at 121°C for 15–30 minutes, then cooled and poured into 100 × 15mm sterile petri dishes. Kanamycin (50 *µ*g/ml) was added to PD3.

Bacteria were inoculated and allowed to grow in liquid medium at 28°C: *Xf* (5 days), *Xa* (3 days), and *Lc* (3 days) to reach the exponential phase. The inoculum was diluted to a working OD of 0.5 (1×10 ^7^ cells/ml). 10 *µ*l of the OD 0.5 was plated with 90 *µ*l of liquid media and spread on the pre-made agar plates to create a confluent lawn of bacteria. The bacteria were given an hour to set at room temperature. 10 *µ*l of each peptide concentration was spotted onto a plate of agar preseeded with a layer of bacterium. After spotting the plates were incubated at 28°C for 2 to 10 days till zones of clearance were clearly visible and the plates were scored for the minimum inhibitory concentration (MIC) as that beyond which no visible clearance was observed. Data presented is in triplicate, and were identical.

## Results

Data used for SCALPEL search methodology to identify plant alpha helical - antimicrobial peptides in the PDB database list.plants.txt: list of PDB IDs resulting from querying the PDB database with the keyword ‘plant’. ALPHAHELICES.zip: DSSP analysis of proteins listed in list.plants.txt to identify alpha helices. RawDataHelix.txt: PAGAL analysis of alpha helices listed in ALPHAHELICES.zip. HTH: Set of all pairs of alpha helices connected with a short (<five residues) loop. Click here for additional data file. Copyright: © 2015 Chakraborty S et al. 2015 Data associated with the article are available under the terms of the Creative Commons Zero "No rights reserved" data waiver (CC0 1.0 Public domain dedication).

### Existing AH-AMPs: the positive controls

**Figure 1.**
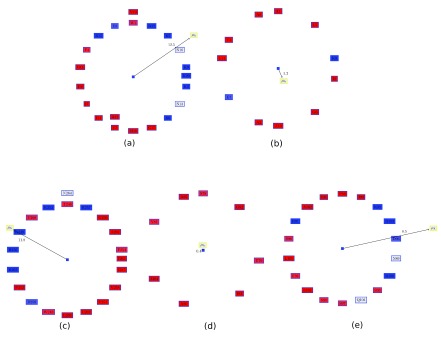
Edmundson wheel for AHs in the known AMPs that were used as control. The color coding for the Edmundson wheel is as follows: all hydrophobic residues are colored red, while hydrophilic residues are colored in blue: dark blue for positively charged residues, medium blue for negatively charged residues and light blue for amides. The hydrophobic moment arrow is not to scale. ( **a**) N-terminal of Cecropin B (CECB) shows its amphipathic nature, with one side being cationic and the other side hydrophobic. ( **b**) C-terminal of CECB consists of mostly hydrophobic residues, and thus has a low hydrophobic moment. ( **c**) Edmundson wheel for PPC20. ( **d**) Edmundson wheel for 3ALGA. *α*4, which corresponds to the C-terminal of CECB and comprises mostly of hydrophobic residues (low hydrophobic moment). ( **e**) Edmundson wheel for 3ALGA. *α*5, which corresponds to the cationic, N-terminal of CECB with a large hydrophobic moment.

Cecropin B (CECB) was used as a positive control, as it is known to target membrane surfaces and creates pores in the bacterial outer membrane ^30, 31^. CECB consists of an cationic amphipathic N-Terminal with a large hydrophobic moment ( Figure 1a), and a C-Terminal comprising mostly of hydrophobic residues, which consequently has a low hydrophobic moment, ( Figure 1b) joined by a short loop. Another positive control was a linear AH-AMP consisting of the residues 2-22 of the N-Terminal in CECB (CBNT21) ( Figure 1a). The sequences of these are shown in Table 1.

**Table 1.** Sequences of peptides used in this study. CO: control peptides SC: SCALPEL generated peptides.

CO	CECB	KWKVFKKIEKMGRNIRNGIVKAGPAIAVLGEAKAL full length CECB from *Hyalophora cecropia* (silk moth)
CO	CBNT21	WKVFKKIEKMGRNIRNGIVKA N-terminal CECB (minus the first lysine)
SC	PPC20	TIWKGVPKFLRRVDTALKNI Linear cationic AH-AMP from phosphoenolpyruvate carboxylase (PDBid:3ZGBA)
SC	CHITI25	TAYGIMARQPNSRKSFIDSSIRLAR CECB like AH-AMP from chitinase *Nicotiana tobaccum* (PDBid:3ALGA)
SC	ISS15	TLDELELFTDAVERW Linear anionic peptide from isoprene synthase from gray poplar (PDBid:3N0FA)

### SCALPEL: Identifying native AH-AMP peptides from the host proteome

**Figure 2.**
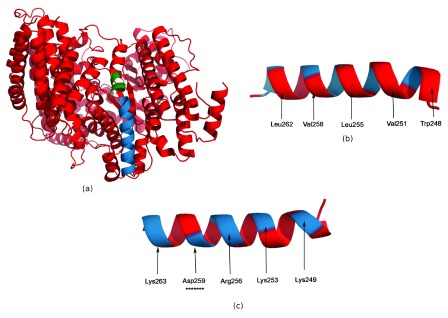
Peptide PPC20 from phosphoenolpyruvate carboxylase in sunflower (PDBid:3ZGBA. *α*11). ( **a**) 3ZGBA. *α*11 is marked in green and blue. We ignore the *π* AH, and also the small AH preceding it (marked in green). PPC20 is marked in blue. ( **b**) Hydrophobic surface of PPC20. ( **c**) Charged surface of PPC20. Asp259 stands out as a negative residue in an otherwise positive surface.

***Linear AH-AMPs***. In order to choose a peptide mimicking CBNT21 (cationic, amphipathic, large hydrophobic moment), we directed our search to ‘locate a small peptide with a large hydrophobic moment and a high proportion of positively charged residues’ on the raw data computed using PAGAL (See RawDataHelix.txt in Dataset 1). A small peptide is essential for quick and cost effective iterations. Table 2 shows the best matching AHs. Next, we used the sequence of these AHs to search the grapevine and citrus genomes, choosing only those that are present in both genomes. This allowed us to locate an AH from phosphoenolpyruvate carboxylase from sunflower, a key enzyme in the C4-photosynthetic carbon cycle which enhances solar conversion efficiency (PDBid:3ZGBA. *α*11) ^33^. Figure 2a shows the specific AH located within the protein structure, marked in green and blue. Although DSSP marks the whole peptide stretch as one AH, we chose the AH in blue due to the presence of a small *π* helix preceding that. We named this peptide PPC20 ( Figure 2, Table 1). This peptide is fully conserved (100% identity in the 20 residues) in both grapevine (Accession id:XP_002285441) and citrus (Accession id:AGS12489.1). Figure 2b,c shows the Pymol rendered AH surfaces of PPC20. The Asp259 stands out as a negative residue in an otherwise positive surface ( Figure 2c). Since previous studies have noted dramatic transitions with a single mutation on the polar face, it would be interesting to find the effect of mutating Asp259 to a cationic residue ^42^.

**Table 2.** Identifying AHs with cationic properties from plant proteins with known structures. All AHs in plant proteins are analyzed using PAGAL, and the data is pruned for AHs with a high proportion of positive residues, and finally sorted based on their hydrophobic moment. The first match is present in both grapevine and citrus (PDBid:3ZGBA. *α*11, which is a phosphoenolpyruvate carboxylase from sunflower). We ignored a small *π* AH in the beginning of this peptide comprising four residues. This peptide has been named PPC20. HM: Hydrophobic moment, RPNR: Relative proportion of positive residues among charged residues, Len: length of the *α*, NCH: number of charged residues.

PDB. *α*	Len	HM	RPNR	NCH
3ZGBA. *α*11 (PPC20)	24	12.6	0.8	8
4HWIA. *α*10	17	12.3	0.9	9
4BXHB. *α*11	23	12.3	0.8	8
2J376. *α*1	18	10.5	0.9	8
3J61R. *α*4	21	10.4	0.9	10
3J60G. *α*3	44	10.2	0.8	22
1W07A. *α*4	21	9.9	0.8	10
2WWBM. *α*1	17	9.5	0.9	8
1B8GA. *α*17	27	7.3	0.9	11
3J61L. *α*1	19	7.2	1	9

**Figure 3.**
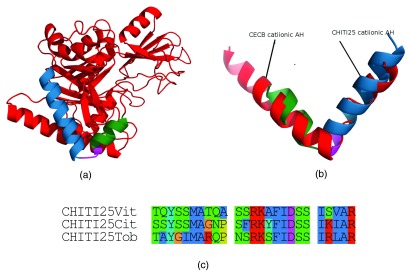
Peptide CHITI25 from chitinase in tobacco (PDBid:3ALGA). ( **a**) PDBid:3ALGA. *α*4 in green, loop in magenta and 3ALGA. *α*5 in blue. ( **b**) Superimposing CECB (PDBid:2IGRA) in red with CHITI25 in green using MUSTANG ^38^. Note, that the order of the AHs are reversed. ( **c**) Multiple sequence alignment of CHITI25 from grapevine (CHITIVit), citrus (CHITICit) and tobacco (CHITITob). CHITITob is the more cationic than CHITIVit or CHITICit.

***Non-linear AH-AMPs consisting of two AHs.*** Next, we located two AHs within chitinase from *Nicotiana tobaccum* (PDBid:3ALGA. *α*4 and 3ALGA. *α*5) ^34^ connected by a short random coil such that one of the AHs is cationic and hydrophobic, while the other AH is comprised mostly of hydrophobic, uncharged residues (CHITI25, Figure 3a, Table 1). This peptide mimics the complete CECB protein ( Figure 3b). While the properties of the AHs in CHITI25 is reversed from that of CECB, the order in which these AHs occur is not important for functionality. The multiple sequence alignment of CHITI25 from grapevine, citrus and tobacco is shown in Figure 3c. CHITI25 from tobacco is the most cationic (five), followed by citrus (four) and grapevine (three). Thus, it is possible that the anti-microbial properties of CHITI25 from grapevine would be lower than CHITI25 from tobacco. These peptides can be subjected to mutations to enhance their natural anti-microbial properties in such a scenario ^43^.

***Negative control - an anionic AH-AMP.*** We also located an anionic AH-AMP using a similar strategy - a 13 residue peptide present within the structure of isoprene synthase from gray poplar (PDBid:3N0FA. *α*18) ^44^. We also used phosphate buffered saline as a negative control. We have extended this helix on both terminals by including one adjacent residue from both terminals to obtain ISS15 ( Table 1).

### *In vitro* results

**Figure 4.**
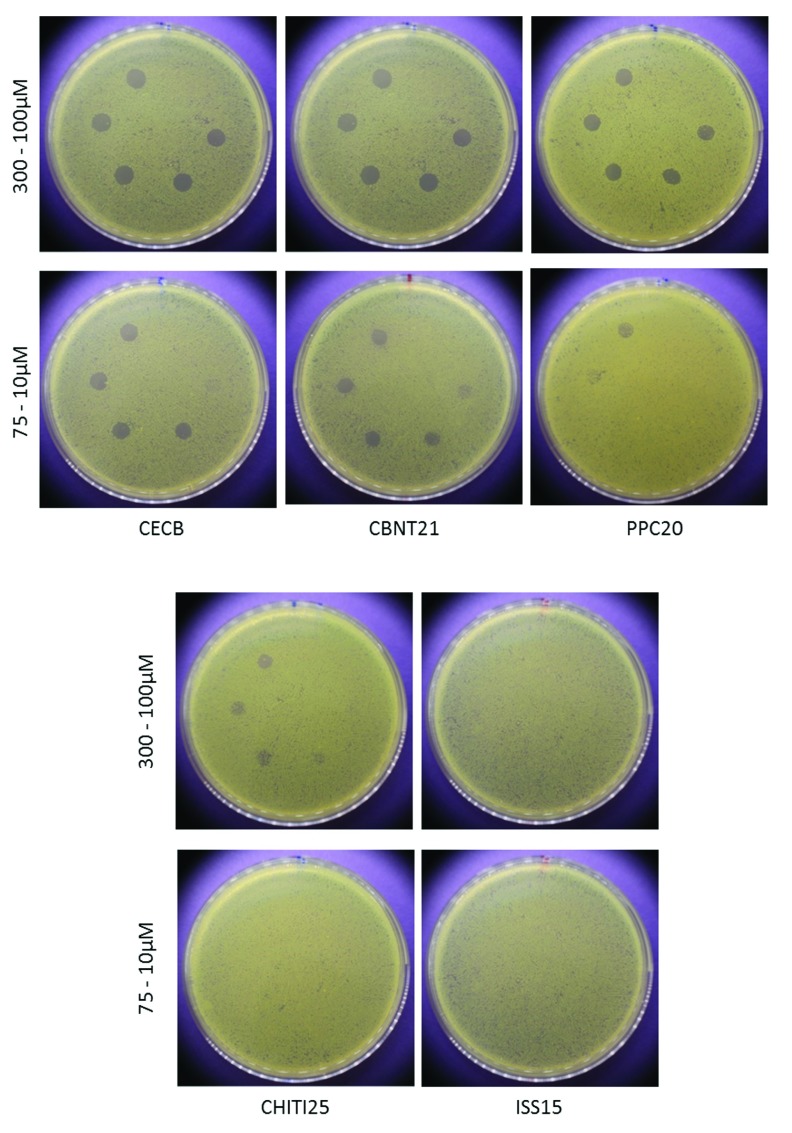
*In vitro* validation of SCALPEL methodology. Plating assay to determine minimum inhibitory concentration (MIC) of SCALPEL identified peptides for *Xanthomonas arboricola*. Counter-clockwise: 300 *µ*M, 250 *µ*M, 200 *µ*M, 150 *µ*M, 100 *µ*M 75 *µ*M, 50 *µ*M, 30 *µ*M, 25 *µ*M, 10 *µ*M, PBS. CECB: MIC 25, CBNT2: MIC 10, PPC20: MIC 50, CHITI25: MIC 150, ISS15: MIC >300.

We have validated our peptides using plating assays ( Table 3, Figure 4). CECB, the well-established AH-AMP, is the most potent among all the peptides tested, having minimum inhibitory concentrations of between 25 *µ*M (for *Xa*) to 100 *µ*M (for *Xf* and *Lc*). This shows the variations in susceptibilities of different organisms. Understanding this differential susceptibility would require a deeper understanding of the underlying mechanism by which these AH-AMPs work ^45^, as well as the difference in the membrane composition of these gram-negative pathogens ^46^. Mostly, CBNT21 has a slightly lower potency, indicating a role for the C-terminal AH in CECB, which comprises of mostly hydrophobic residues for *Xf* and *Lc*. This results corroborates a plausible mechanism suggested by others in which the anionic membranes of bacteria is targeted by the cationic N-terminal, and followed by the insertion of the C-terminal AH into the hydrophobic membrane creating a pore. PPC20 and CHITI25 have comparable potencies with CECB and CBNT21, although *Lc* appears to be resistant to CHITI25. Finally, the anionic peptide used as a negative control shows no effect on these pathogens.

**Table 3.** Minimum Inhibitory Concentration of peptides tested ( *µ*M). It can be seen that CECB is the most efficient among all the peptides for all three pathogens, while the anionic ISS15 does not show any effect even at higher concentrations. However, while CHITI25 is almost as effective as CECB for *Xf*, it fails to inhibit *Lc* growth. Also, *Xa* is much more susceptible to these peptides compared to the other two pathogens. Finally, the anionic ISS15 has no effect on these pathogens. Data is in triplicate, and were identical.

	Bacteria	CECB	CBNT21	PPC20	CHITI25	ISS15
*γ* Proteobacteria	*Xylella fastidiosa* 3A2 ( *Xf*) *Xanthomonas arboricola* 417 ( *Xa*)	100 25	200 25	150 50	100 150	>300 >300
*α* Proteobacteria	*Liberibacter crescens* BT-1 ( *Lc*)	100	200	200	>300	>300

## Discussion

The repertoire of defense proteins available to an organism is being constantly reshaped through genomic changes that confer resistance to pathogens. Genetic approaches aim at achieving the same goal of enhancing immunity through rational design of peptides ^13, 47^, which are then incorporated into the genome ^29, 31, 48^. Also, it is important to ensure that these non-endogenous genomic fragments have minimal effect on humans for their commercial viability ^32^. Identifying peptides from the same genome helps allay these concerns to a significant extent. The key innovation of the current work is the ability to identify peptides with specific properties (cationic AHs with a hydrophobic surface, linear or otherwise) from the genome of any organism of interest. Such peptides also present less likelihood of eliciting an adverse immune response from the host.

### Alternate methods

Alternate computational methods for finding such new AMPs based on known AMPs could be of two kinds, although neither method is as effective in obtaining our results. Firstly, a sequence search using BLAST can be done to find a corresponding peptide in the genome, say for cecropin B. However, a BLAST of the cecropin sequence does not give any significant matches in the grapevine or citrus genomes, and is a dead end. In principle, what we need is a peptide with cecropin B like properties - and that information is not encoded in the linear sequence, but in the Edmundson wheel of the AH. The second method for such a search is to find structural homology in the PDB database through a tool like DALILITE ^49^. However, AHs are almost indistinguishable structurally, and the results will give rise to many redundancies. Thus, there are no existing methods tailored to incorporate the quantifiable properties of AHs in the search. We, for the first time, have proposed such a method in SCALPEL.

Computer-assisted design strategies have also been applied in designing *de novo* AMPs ^50, 51^. Other hand curated comprehensive databases for ‘for storing, classifying, searching, predicting, and designing potent peptides against pathogenic bacteria, viruses, fungi, parasites, and cancer cells’ ^52^ do not enjoy the automation and vastness of available data elucidated in the SCALPEL methodology.

### Limitations and future directions

There are several caveats to our study. We are yet to ascertain the hemolytic nature of the identified peptides, and will be performing these experiments in the near future. In fact, the selective cytotoxicity against human cancer cells, might be used as a substitute therapy in place of conventional chemotherapy ^53, 54^. It must be noted that the development of a selective peptide with anti-cancer cell properties has been a challenge ^55^. Although, we have not measured the lipid permeabilizing abilities of our peptides, a recent study has found that potency in permeabilizing bacteria-like lipid vesicles does not correlate with significant improvements in antimicrobial activity, rendering such measurements redundant ^56^. The electrostatic context of an peptide is known to have a significant bearing on its propensity to adopt an AH structure. The ability to predict the folding of peptides requires significant computational power and modelling expertise ^57^. Peptides often remain in random coil conformations, and achieve helical structures only by interacting with anionic membrane models ^58^. It is also possible to measure peptide helicity through circular dichroism spectroscopy ^59^. However, our results have been all positive based on selected choices of peptides arising from our search results, and suggest a high likelihood of getting anti-microbial activity from these peptides. Additionally, we may have to resort to other innovative techniques that have been previously adopted to overcome thermodynamic instability or proteolytic susceptibility ^60– 63^.

## Conclusion

To summarize, we establish the presence of a large number of AH-AMPs ‘hidden’ in the universal proteome. We have designed a methodology to extract such peptides from the PDB database - the ‘Big Data’ center in proteomics. We demonstrate our results on well known plant pathogens - *Xf, Xa* and *Lc*. The feasibility of using such peptides in cancer therapies is also strong ^54, 64^. The ability to choose a peptide from the host itself is an invaluable asset, since nativeness of the peptide allays fears of eliciting a negative immune response upon administration. The problem of antibiotic resistance is also increasing focus on peptide based therapies ^9, 65^, since it is ‘an enigma that bacteria have not developed highly effective cationic AMP-resistance mechanisms’ ^66^. Lastly, in face of the current Ebola outbreak ^67, 68^, we strongly suggest the possibility of developing peptides derived from the human genome to target viral epitopes, such as those enumerated for the Ebola virus recently ^7^. A recent study has reported the inhibition of the Ebola virus entry and infection by several cationic amphiphiles ^69^, suggesting the SCALPEL generated cationic peptides with the aid of cell penetrating peptides ^70^ could achieve similar results.

## Data Availability

F1000Research: Dataset 1. Data used for SCAPEL search methodology to identify plant alpha helical - antimicrobial peptides in the PDB database, 10.5256/f1000research.5802.d39823 ^71^
