# Supplementary material for: The PDB database is a rich source of alpha-helical anti-microbial peptides to combat disease causing pathogens
Source: F1000Res. 2015 Jun 16;3:295. Originally published 2014 Dec 5. [Version 2] doi: 10.12688/f1000research.5802.2 (PMC4642847; doi:10.12688/f1000research.5802.2)
Supplement: Peer review history

# Peer review: Referee response for version 2

**DOI:** 10.5256/f1000research.7108.r11126

**Published:** 2015-11-06

**Authors:** Kevan Hartshorn

**Affiliations:** 1 Department of Medicine, Boston University, Boston, MA, USA

I think this article is quite interesting and think the authors have answered prior critiques reasonable well. I do not have much in the way of further critiques.

My specific critiques are as follows:

1. The results presented in Table 3 do not show any statistics (e.g. p values or standard error).

2. The best negative control for such studies is use of a scrambled version of peptide.

I have read this submission. I believe that I have an appropriate level of expertise to confirm that it is of an acceptable scientific standard.

# Peer review: Referee response for version 2

**DOI:** 10.5256/f1000research.7108.r10406

**Published:** 2015-10-19

**Authors:** Autar Mattoo, Ravinder Goyal

**Affiliations:** 1 Henry A. Wallace Beltsville Agricultural Research Center, United States Department of Agriculture, Beltsville, MD, USA; 2 AAFC Lethbridge Research Center, Alberta, Canada

**Title:**

Suggest inserting 'sequences' after anti-microbial peptide, and include 'plant' since the search was made for plant proteins and validated on phytopathogens.

**Abstract:**

The contents starting from “Substitute therapies .... end”  are hypothetical and need to be deleted or toned down. 

**Introduction:**

Paragraph 3. Clarify which humanitarian reasons are associated with the listed plant pathogens. The central theme of the manuscript is a new search algorithm, SCALPEL and its validation. Therefore, there is a need for reasoning the development of a new algorithm in light of the existing software(s), if any. In other words, what is the necessity of a new algorithm? The introduction is not crisp and balanced. 

**Materials and Methods: **

- *In silico:* Is their a need to list all 2000 proteins in Dataset identified using search ‘plants’. Simply, it could be stated that as many plant proteins were analyzed. Need more details for SCALPEL vis-á-vis methodology.
- *In vitro:* Why was Kanamycin added to PD3?

**Results:**

1. Figure 1a: An explanation is required for omitting the first ‘K’ in Edmundson wheel.
2. The main legend to Figure 1 “Edmundson wheel for AHs in the known AMPs that were used as control” is confusing. It does not look like the peptides corresponding to wheels c, d and e were the controls.
3. PPC20, 3ALGA.α4 and 3ALGA.α5 (Fig. 1c, d, e) should be described when first mentioned in the text.
4. Non-linear AH-AMPs consisting of two AHs: “While the properties of the AHs in CHITI25 is reversed from that of CECB, the order in which these AHs occur is not important for functionality” is an overstatement unless proved.
5. Figure 4. The PBS used for peptide dilution does not appear as a control in the Figure. The control ISS15 does not show pathogen growth inhibition at the maximum concentration tested. Thus, to conclude its MIC is >300 microM is a speculation.

**Discussion:**

The discussion is entirely focused on the search software that is hardly mentioned in the Introduction, Methodology, and Results. There is more focus on the application of the current study instead of the study itself. The readership would benefit more if the discussion included scrutiny and reaffirmation of the results with relevant literature and interpretation.

We have read this submission. We believe that we have an appropriate level of expertise to confirm that it is of an acceptable scientific standard, however we have significant reservations, as outlined above.

# Peer review: Referee response for version 2

**DOI:** 10.5256/f1000research.7108.r8667

**Published:** 2015-06-17

**Authors:** Jean-Marc Berjeaud

**Affiliations:** 1 Laboratory of Ecological and Biological Interactions, University of Poitiers, Poitiers, France

The corrections made in the new version are in agreement with my requests. As a consequence I approve the indexing of this article.

I have read this submission. I believe that I have an appropriate level of expertise to confirm that it is of an acceptable scientific standard.

# Peer review: Referee response for version 1

**DOI:** 10.5256/f1000research.6202.r8820

**Published:** 2015-06-01

**Authors:** Jean-Marc Berjeaud

**Affiliations:** 1 Laboratory of Ecological and Biological Interactions, University of Poitiers, Poitiers, France

***Title and Abstract***:

The Title of the article, "The PDB database is a rich source of alpha-helical anti-microbial peptides to combat disease causing pathogens", is appropriate for the content of the article. However because the detected peptides were solely tested toward plant pathogens the term of "plant" in the title could be considered. The abstract represent rather well the work presented in the article except the two last sentences which are expectations of the authors but were not studied in this work. Particularly the sentence : "The use of native…" assert that peptide structure extracted from native proteins will be without adverse effect to the host but it has to be proved in my opinion.

***Article content***:

The paper describes the use of a software and an *in silico *method developed by the authors to screen the protein database (PDB) to find new antimicrobial peptides on the basis of the secondary structures of these peptides. The method is innovative and very interesting. Moreover the authors proved the efficacy of their method as they synthesized peptides from portions of protein sequences presenting secondary structures resembling cecropin B and demonstrated the antimicrobial activity of these new peptides.

However I did not understand why they used an anionic peptide as the negative control. Indeed it is well known that the global positive charge of the peptides is required for their initial stacking on the membrane of target cells. Thus it is predictable that any anionic peptide will be inactive.

***Conclusions***:

The main problem concerns the conclusions of the article. Indeed there are not sufficient experimental evidences in the article to assert that the alpha-helical peptides with antimicrobial activity have a strong potency to act toward cancer cells. In my experience I tested several alpha-helical peptides which were solely antimicrobial. Thus I strongly suggest to moderate this conclusions part of the manuscript.

I have read this submission. I believe that I have an appropriate level of expertise to confirm that it is of an acceptable scientific standard.

# Response

**Published:** 2015-06-04

**Authors:** Sandeep Chakraborty

**Affiliations:** Tata Institute of Fundamental Research, India

Dear Dr Berjeaud,

We would like to thank you for taking the time to review this paper. Please find our responses below.

**Title and Abstract: The Title of the article, ”The PDB database is a rich source of alpha-helical anti-microbial peptides to combat disease causing pathogens”, is appropriate for the content of the article. However because the detected peptides were solely tested toward plant pathogens the term of ”plant” in the title could be considered.**

We are in the process of testing these peptides on other pathogens. Furthermore, considering that the mechanism of the inhibitory effect of these peptides is independent of the pathogen host, we are quite confident of replicating our results on other ‘non-plant’ pathogens.

**The abstract represent rather well the work presented in the article except the two last sentences which are expectations of the authors but were not studied in this work. Particularly the sentence : ”The use of native” assert that peptide structure extracted from native proteins will be without adverse effect to the host but it has to be proved in my opinion.**

We believe this is an important hypothesis, although unproven, which differentiates SCALPEL from other methods of identifying anti-microbial peptides. However, we have modified the statement in the abstract to make this less of an assertion, and more of a hypothesis.

**Article content: The paper describes the use of a software and an in silico method developed by the authors to screen the protein database (PDB) to find new antimicrobial peptides on the basis of the secondary structures of these peptides. The method is innovative and very interesting. Moreover the authors proved the efficacy of their method as they synthesized peptides from portions of protein sequences presenting secondary structures resembling cecropin B and demonstrated the antimicrobial activity of these new peptides.**

We appreciate the positive comments.

**However I did not understand why they used an anionic peptide as the negative control. Indeed it is well known that the global positive charge of the peptides is required for their initial stacking on the membrane of target cells. Thus it is predictable that any anionic peptide will be inactive.**

We have used this as a negative control. If our experimental setup in the process of adding the peptides had any undesired conditions which was inhibiting the pathogens, this anionic peptide would show positive results. So, this is slightly different from a null negative control, as it involves adding a peptide.

**Conclusions: The main problem concerns the conclusions of the article. Indeed there are not sufficient experimental evidences in the article to assert that the alpha-helical peptides with antimicrobial activity have a strong potency to act toward cancer cells. In my experience I tested several alpha-helical peptides which were solely antimicrobial. Thus I strongly suggest to moderate this conclusions part of the manuscript.**

We appreciate your concern regarding the lack of confirmatory evidence of AH peptides as anti-cancer therapeutics. However, this continues to be an active front in research, and we hope that SCALPEL will provide further avenues for testing this hypothesis. We have cited two recent papers- http://www.ncbi.nlm.nih.gov/pubmed/25270878 and http://www.ncbi.nlm.nih.gov/pubmed/24101917

in this context. Also, we have modified the text to reflect the lack of conclusiveness in such studies.Once again, we are thankful for your insightful comments, and hope to have addressed your concerns.

Thanking you,

Sincerely,

Sandeep Chakraborty

Plant Sciences Department,

University of California, Davis, CA 95616, USA.
